# Injectable and oral contraceptives and risk of HIV acquisition in women: an analysis of data from the MDP301 trial

**DOI:** 10.1093/humrep/deu113

**Published:** 2014-05-16

**Authors:** Angela M. Crook, Deborah Ford, Mitzy Gafos, Richard Hayes, Anatoli Kamali, Saidi Kapiga, Andrew Nunn, Maureen Chisembele, Gita Ramjee, Helen Rees, Sheena McCormack

**Affiliations:** 1 MRC Clinical Trials Unit at UCL, London, UK; 2 Africa Centre for Health and Population Studies, Mtubatuba, South Africa; 3 London School of Hygiene and Tropical Medicine, London, United Kingdom; 4 MRC/UVRI Uganda Research Unit, Entebbe, Uganda; 5 Mwanza Intervention Trials Unit (MITU), Mwanza, Tanzania; 6 University Teaching Hospital, Lusaka, Zambia; 7 MRC HIV Prevention Research Unit, Durban, South Africa; 8 Wits Reproductive Health and HIV Institute, University of the Witwatersrand, Johannesburg, South Africa

**Keywords:** HIV, contraception, women, sexual behaviour

## Abstract

**STUDY QUESTION:**

Do injectable and oral contraceptives increase the risk of human immunodeficiency virus (HIV) acquisition in women?

**SUMMARY ANSWER:**

After adjusting for confounders, evidence of a significantly increased risk of HIV remained for women using injectable depo-medroxyprogesterone (DMPA) (hazard ratio = 1.49, 95% confidence interval (1.06–2.08)) but not for injectable norethisterone-enanthate (Net-En) or oral contraceptive pills (OC).

**WHAT IS KNOWN ALREADY:**

An association between the use of some types of hormonal contraception (HC) methods and an increased risk of HIV, possibly through changes in the genital tract environment and alterations in the immune response, has been previously observed, although not consistently. A recent systematic review of these studies has highlighted the need for more definitive evidence.

**STUDY DESIGN, SIZE, DURATION:**

A secondary data analysis of the MDP301 phase 3 microbicide trial was conducted to estimate the effects of use of different methods of HC on the risk of HIV acquisition in women. HIV-negative women (*n* = 8663) with a median age of 28 years were included in the analysis; 382 HIV seroconverted by 52 weeks follow-up; 10% of women-years were lost to follow-up before 52 weeks.

**PARTICIPANTS/MATERIALS, SETTING, METHODS:**

Contraceptive use was reported at each 4-weekly visit. Cox proportional hazards (PH) models were used to estimate the effects of baseline and current use of injectable DMPA, injectable Net-En and OC compared with no HC, on the risk of HIV, adjusting for baseline and time-updated covariates. Causal effects for 52 weeks of HC use compared with no HC were estimated in a weighted Cox model, censoring women at deviation from baseline HC use (or non-use) or pregnancy.

**MAIN RESULTS AND THE ROLE OF CHANCE:**

At baseline, 2499 (29%) women were on DMPA, 1180 (14%) on Net-En, and 1410 (16%) on OC; 3574 (40%) not on HC, started HC in follow-up. Adjusted hazard ratios (HR) for baseline HC use, compared with no HC, were 1.38 (95% confidence interval (CI) 1.07–1.78) for DMPA; 1.18 (0.86–1.62) for Net-En and 0.97 (0.68–1.38) for OC. The estimated causal effects of DMPA and Net-En over 52 weeks were: HR = 1.49 (95% CI 1.06–2.08) and HR = 1.31 (95% CI 0.62–1.61), respectively.

**LIMITATIONS, REASONS FOR CAUTION:**

A main limitation of the study was that it was a secondary analysis of data from a study that was not designed to investigate this question. Despite our best efforts, we cannot exclude residual confounding to explain the effect of DMPA.

**WIDER IMPLICATIONS OF THE FINDINGS:**

The results of this study should be reviewed by the World Health Organization to determine whether current recommendations on the use of DMPA in settings with high HIV prevalence require modification.

**STUDY FUNDING/COMPETING INTEREST(S):**

MDP is a partnership of African and European academic/government institutions with commercial organizations, which is funded by the UK Government (DFID and MRC), with support from IPM and EDCTP. The funders had no role in study design, data collection and analysis, decision to publish, or preparation of the manuscript. Competing interests: None.

## Introduction

A number of epidemiological studies have investigated the relationship between the use of different oral and injectable hormonal contraception (HC) and possible increased risk of human immunodeficiency virus (HIV) infection. A recent systematic review of these has highlighted the need for more definitive evidence (Polis and Curtis, 2013). There are possible biological mechanisms which might explain a potential elevated risk, including changes in the genital tract environment and alteration of immune response (Marx et al*.*, 1996; Trunova *et al.*, 2006; Hel *et al.*, 2010). In early 2012, the World Health Organization (WHO) issued a technical statement following a review of the available evidence. While they did not propose any restrictions on the use of hormonal contraception (HC), for women at high risk of HIV they added a clarification to their guidance stating: ‘because of the inconclusive nature of the body of evidence on possible increased risk of HIV acquisition, women using progestogen-only injectable contraception should be strongly advised to also always use condoms, male or female, and other HIV preventive measures.’ (WHO, 2012).

In the absence of any RCT of HC methods and HIV acquisition in women, observational data from HIV prevention trials provide an opportunity to investigate this question. We aimed to estimate the effect of HC use on the risk of HIV acquisition using a large dataset drawn from the MDP301 phase 3 microbicide trial. Data came from six African sites with high HIV incidence and included 4-weekly collection of data on contraception to 52 weeks follow-up after enrolment.

Note that we use the term ‘hormonal contraception’ to mean injectable and oral contraceptives.

## Methods

### Data

The trial design, methods and results have been described elsewhere (Nunn *et al.*, 2009; McCormack *et al.*, 2010; Pool *et al.*, 2010a,b). Briefly, MDP301 was a phase 3 double blind randomized placebo-controlled trial investigating the safety and efficacy of the candidate microbicide PRO2000, formulated in a gel and inserted prior to sex via a vaginal applicator. Trial registration: ISRCTN64716212. A total of 9385 women were enrolled from six centres from four sub-Saharan African countries: three centres in South Africa and one each in Uganda, Tanzania and Zambia. HIV-negative women aged 16 or older (age 18 years or older in South Africa and Zambia) were recruited in a range of settings and in Uganda were recruited as sero-discordant couples with their HIV-positive partner. The primary end-point was incidence of HIV infection over 52 weeks of follow-up and the study concluded that there was no evidence that PRO2000 gel protected women from HIV infection. A total of 8663 HIV-negative women were included in the present analysis. Those excluded were: 69 participants found to be HIV positive at enrolment, 458 who had no follow-up data on HIV and a further 18 using hormonal implants who were considered too few to form a separate group for analysis. In addition 177 participants aged 51 years or above were also excluded as being beyond reproductive age. Participants reported contraception at baseline and then at each 4-weekly visit through to Week 52.

### Outcome

During the 52 weeks follow-up, the 8663 study participants contributed a total of 8122 person-years (py) for analysis. HIV testing occurred at Weeks 12, 24, 40 and 52 and results were confirmed in a central laboratory (Jentsch *et al.*, 2012). Follow-up time was measured from enrolment to the date of the earliest positive HIV test, last follow-up visit or Week 52. A total of 382 incident HIV infections were included in the analysis.

### Exposure

Data on contraception were collected every 4 weeks from enrolment from the following question in the sexual behavioural case record form: ‘Are you currently using any form of family planning?’ Where available, these data were cross-checked with the contraception dispensing data. Responses for type of HC were categorized for analysis as: (i) injectable depo-medroxyprogesterone (DMPA) (ii) injectable norethisterone enanthate (Net-En); (iii) oral contraceptive pills (OC) and (iv) not using HC (the reference category). This reference category was chosen to aid comparison with previous studies and included the following types of contraception: no contraception (44% at baseline); condoms for family planning (male or female) (50% at baseline); natural or traditional methods (e.g. wooden band or local herbs) (4% at baseline); sterilization (1% at baseline); intrauterine contraceptive device (1% at baseline).

Note that data collected on OC did not distinguish between different types of pill. In the setting of this study however it was likely that the combined OC would have been used.

### Covariates

Baseline variables considered were: socio-demographic (age, centre, education, employment status, randomized gel group); sexual behaviour (age of first sexual intercourse, sexual frequency during the last 7 days, condom use at last sex act, anal sex in last 4 weeks, sex during menses and number of sexual partners (reported in the last 4 weeks)); reported genital history (unusual discharge, itching, ulcers and ectopy); and laboratory findings (described below). Covariates measured in follow-up were: sexual frequency during the last 7 days and condom use at last sex act (4-weekly); genital findings reported every 4 weeks; urine tests for pregnancy taken 4-weekly from enrolment; herpes simplex virus (HSV)-2 serology (for those HSV-2 seronegative at enrolment) and confirmed by the central laboratory at Weeks 0, 40 and 52; presence of *Neisseria gonorrhoea* (NG), or *Chlamydia trachomatis* determined from an endocervical swab taken at Weeks 0 and 24; *Trichomonas vaginalis* diagnosed using the In Pouch culture method also at Weeks 0 and 24; syphilis determined serologically at Weeks 0, 40 and 52; bacterial vaginosis (Ison Hay) at Weeks 0, 12, 24, 40 and 52. Additional tests were performed at unscheduled visits if indicated.

All covariates (including contraception) measured in follow-up were taken from the last reported visit. For missed visits or at visits where tests were not scheduled, we carried forward the value from the previous visit. A sensitivity analysis was performed to assess the effect of missed visits.

### Statistical methods

Cox proportional hazards models were used to estimate the hazard ratios for HIV acquisition associated with DMPA, Net-En and OC compared with no HC. The parameters of the Cox models were estimated by pooled logistic regression models, modelling the change in baseline hazard since enrolment using a restricted cubic spline with knots at the 10th, 50th and 90th percentiles (Weeks 8, 28 and 48). Data were organized into 4-weekly intervals, corresponding to the visit schedule. Time-dependent variables were the most recent values up to the start of each interval. Separate models were fitted for the effect of *baseline* use of contraception and *current* use of contraception on risk of HIV acquisition adjusting for baseline and time-updated covariates. A limitation of the *baseline* model is that it does not account for starting, stopping or changing type of HC during the study; a limitation of the *current* model is that it does not appropriately adjust for time-dependent confounders which may be affected by past HC exposure (although marginal structural models and Cox PH models have given similar results in previous studies (Heffron *et al.*, 2012; McCoy *et al.*, 2013)). To attempt to estimate the causal hazard ratio of HC use on HIV risk over 52 weeks, compared with 52 weeks of no HC use in non-pregnant women we used a weighted Cox proportional hazards model. Women were ‘artificially censored’ at their first change in baseline HC or non-use of HC (starting, stopping or switching type) or first positive pregnancy test. The association between baseline HC and HIV diagnosis was estimated using inverse-probability-of-censoring-weights to account for artificial censoring, and direct adjustment for baseline covariates to adjust for predictors of baseline HC group (Robins *et al.*, 2000; Hernan *et al.*, 2006; Cole and Hernan, 2008). While some information is lost (artificial censoring, including censoring for pregnancy, resulted in 100 HIV infections and 23% of person-years being excluded from the analysis), this model corrects for starting, stopping, changing HC type during follow-up and allows for measured time-dependent confounders appropriately. It has the advantage over a standard MSM in that the type of contraception used at the time of HIV infection (for included outcomes) is known, as data are censored at first change in HC and HIV infection is at the first positive HIV test. Full details on the casual model are given in the Supplementary Data.

The following sensitivity analyses were also performed and were conducted on the final adjusted *baseline* HC model and *current* HC model on the full dataset. A previous study showed age and HSV-2 to be significant effect modifiers, with larger effects of HC found for women using HC who were under 25 years or who were HSV-2 negative at enrolment (Morrison *et al.*, 2010) and another study also showed effect modification for age (Morrison *et al.*, 2012). Therefore, age (<25 years versus ≥25 years), HSV-2 status at enrolment and centre (to test for consistency of the results between the different trial populations) were investigated as effect modifiers by tests for interaction. It should be noted that other studies have failed to find evidence of effect modification (Heffron *et al.*, 2012; McCoy *et al.*, 2013).

There is potential differential HIV risk behaviour within the no HC group, with women using condoms for contraception likely to use condoms more consistently (compared with those using other non-hormonal methods) and hence to be at lower risk of HIV infection. We therefore conducted an analysis separating out those in the no HC group who reported using condoms as their main method of contraception, as a separate group.

Finally to exclude any possible effect of long-term cumulative exposure to HC, we repeated the analysis restricted to those who were not on HC at the screening visit (which occurred up to 6 weeks before enrolment).

All analyses were performed in Stata (version 12.1). A value of *P*< 0.05 was considered significant.

## Results

### Baseline use of HC

Baseline characteristics of study participants by use of HC at baseline are shown in Table I: 2499 (29%) women were on DMPA, 1180 (14%) on Net-En, 1410 (16%) on OC and 3574 (41%) on no HC at enrolment. There were notable differences in HC use between centres, with injectable HC use more common in the South African sites, and the majority of Net-En users enrolled through Johannesburg. OC was more common in Zambia. Younger women were more likely to be prescribed HC, particularly Net-En. Net-En users were more likely to have had secondary education and less likely to be employed. Net-En users were also more likely to report using a condom at the last sex act and having sex during menses. Notably OC users were less likely to report using a condom at the last sex act at baseline. Women on no HC were more likely to report not having sex the week prior to enrolment and were more likely to report a history of genital symptoms and to have positive laboratory findings for some sexually transmitted infections.

**Table I:** Baseline characteristics of women by hormonal contraception (HC) use at enrolment.

Characteristic	Injectable DMPA (*n* = 2499)	Injectable Net-En (*n* = 1180)	OC (*n* = 1410)	No HC (*n* = 3574)	Total *N* = 8663	*P*
Trial site						
Durban, SA	972 (39)	297 (25)	195 (14)	853 (24)	2317 (27)	<0.001
Johannesburg, SA	417 (17)	731 (62)	317 (22)	895 (25)	2360 (27)	
Africa Centre, SA	356 (14)	82 (7)	69 (5)	508 (14)	1016 (12)	
Masaka, Uganda	277 (11)	0 (0)	55 (4)	454 (13)	786 (9)	
Mwanza, Tanzania	238 (13)	0 (0)	186 (13)	511 (14)	1025 (12)	
Mazabuka, Zambia	149 (6)	69 (6)	588 (42)	353 (10)	1159 (13)	
Socio-demographic						
Age (mean (SD))	28.0 (7.4)	24.7 (6.0)	28.3 (7.2)	32.4 (9.4)	29.9 (9.5)	
16–24 years	1022 (41)	718 (61)	533 (38)	990 (27)	3263 (37)	
25–34 years	957 (38)	355 (30)	582 (41)	1044 (29)	2938 (34)	<0.001
35–44 years	465 (19)	101 (9)	267 (19)	1056 (30)	1889 (22)	
45–50 years	55 (2)	6 (1)	28 (2)	484 (14)	573 (7)	
Education						
None/primary	1948 (78)	674 (57)	1116 (79)	2803 (78)	6541 (76)	<0.001
Secondary/tertiary	551 (22)	506 (43)	294 (21)	771 (22)	2122 (24)	
Employed	621 (25)	151 (13)	334 (24)	972 (27)	2078 (24)	<0.001
Sexual behaviour						
Age of first sex						
<14 years	177 (7)	38 (3)	119 (9)	260 (7)	594 (7)	<0.001
17–20 years	2010 (81)	1021 (87)	1067 (77)	2788 (78)	6886 (80)	
>21 years	307 (12)	118 (10)	204 (15)	507 (14)	1136 (13)	
Sexual frequency^a^ (median (IQR))	2 (1–3)	2 (1–4)	2 (1–4)	2 (1–3)	2 (1–3)	<0.001
No sex in last 7 days	454 (18)	158 (13)	193 (14)	822 (23)	1627 (19)	<0.001
Condom at last sex act	1404 (56)	795 (68)	549 (39)	2228 (62)	4976 (57)	<0.001
Anal sex	37 (1)	9 (1)	16 (1)	32 (1)	94 (1)	0.115
Sex during menses	70 (3)	87 (8)	73 (5)	126 (4)	356 (4)	<0.001
Genital findings						
Discharge (history)	633 (25)	257 (22)	268 (19)	970 (27)	2128 (25)	<0.001
Itching (history)	608 (24)	159 (14)	309 (22)	992 (28)	2068 (24)	<0.001
Ulcers (history)	280 (11)	66 (5)	104 (7)	458 (13)	908 (10)	<0.001
Ectopy, ≥1% (exam)	436 (17)	206 (17)	218 (15)	421 (12)	1281 (15)	<0.001
Positive laboratory test findings						
HSV-2	1614 (66)	567 (50)	855 (62)	2438 (70)	5474 (65)	<0.001
*Chlamydia trachomatis*	246 (10)	158 (14)	78 (6)	200 (6)	682 (8)	<0.001
*Neisseria gonorrhoea*	108 (4)	39 (3)	29 (2)	108 (3)	284 (3)	0.002
Syphilis	89 (4)	29 (2)	42 (3)	164 (5)	324 (4)	0.002
*Trichomonas vaginalis*	207 (8)	94 (8)	104 (7)	409 (12)	814 (9)	<0.001
Bacterial vaginosis	807 (32)	409 (35)	494 (35)	1420 (40)	3130 (36)	<0.001

Values are *n* (%) unless otherwise stated; *P*-values are from Chi-square tests or one-way analysis of variance as appropriate.

DMPA, depo-medroxyprogesterone; Net-En, norethisterone-enanthate; OC, oral contraceptive pill; IQR, inter-quartile range; HSV-2, herpes simplex virus.

^a^During last 7 days.

### Changing HC use in follow-up

Table II describes the changing use of HC during the study. Overall, 64% remained on their baseline contraception in follow-up. Of the 3574 women who were on no HC at baseline, 2130 (60%) remained so in follow-up, while 1444 (40%) started HC during follow-up (449 DMPA, 227 Net-En and 768 OC). Of the 5089 women who started on HC, 3407 (67%) remained on the same type of HC in follow-up.

**Table II:** HC use at baseline and follow-up.

	Contraception at enrolment			
	Injectable DMPA (*n* = 2499)	Injectable Net-En (*n* = 1180)	Oral contraception (*n* = 1410)	No hormonal contraception (*n* = 3574)
Contraception during follow-up				
No change from baseline	1766 (71)	751 (64)	890 (63)	2130 (60)
First change in follow-up				
Started HC	–	–	–	1444 (40)
Switched HC type	421 (17)	267 (23)	236 (17)	–
Stopped HC	312 (14)	162 (14)	284 (20)	–

Values are *n* (%).

### HIV incidence by HC use at enrolment

Table III shows HIV incidence by HC at baseline. A total of 382 HIV infections resulted in HIV incidence of 6.2 per 100 py for baseline DMPA users; 6.3 per 100 py for Net-En users, 3.8 per 100 py for OC users and 3.5 per 100 py for no HC users. Incidence was highest in Durban, South Africa and lowest in Mwanza, Tanzania; it was highest in the under 25 year age group and for those who tested positive for HSV2 at baseline. The proportion of women lost to follow-up (prior to HIV seroconversion) was similar across baseline HC groups: 9, 10, 11 and 10% for DMPA, Net-En, OC and No HC, respectively.

**Table III:** Incidence of human immunodeficiency virus (HIV) by HC (at enrolment).

	DMPA (*n* = 2499)		Net-En (*n* = 1180)		Oral contraception (*n* = 1410)		No hormonal contraception (*n* = 3574)		Total *N* = 8663	
Overall^a^	146/2341	**6.2** (**5.3**–**7.3)**	69/1102	**6.3** (**4.9–7.9)**	50/1321	**3.8** (**2.9–5.0)**	117/3358	**3.5** (**2.9–4.2)**	382/8122	**4.7** (**4.3–5.2)**
% person-years lost										
Trial sites										
Durban	62/920	6.7 (5.3**–**8.6)	29/279	10.4 (7.3**–**15.0)	9/186	4.8 (2.5**–**9.3)	33/821	4.0 (2.9**–**5.6)	133/2206	6.0 (5.1**–**7.1)
Johannesburg	27/389	7.0 (4.8**–**10.2)	32/683	4.7 (3.3**–**6.6)	9/300	3.0 (1.6**–**5.8)	40/819	4.9 (3.6**–**6.71)	108/2190	4.9 (4.1**–**6.0)
Africa Centre	22/329	6.7 (4.4–10.2)	6/76	7.9 (3.5–17.6)	6/63	9.5 (4.2–21.0)	10/481	2.1 (1.1–3.9)	44/950	4.6 (3.4–6.2)
Masaka	19/259	7.3 (4.7–11.5)	0	–	4/51	7.8 (2.9–20.1)	15/435	3.4 (2.1–5.7)	38/746	5.1 (3.7–7.0)
Mwanza	6/312	1.9 (0.9–4.3)	0	–	3/178	1.7 (0.5–5.2)	6/476	1.3 (0.6–2.8)	15/965	1.6 (0.9–2.6)
Mazabuka	10/133	7.5 (4.0–13.9)	2/64	3.1 (0.8–12.5)	19/543	3.5 (2.2–5.5)	13/324	4.0 (2.3–6.9)	44/1064	4.1 (3.1–5.6)
Age group										
<25 years	84/939	8.9 (7.2–11.1)	47/668	7.0 (5.3–9.4)	25/490	5.1 (3.5–7.6)	59/889	6.6 (5.1–8.6)	215/2986	7.2 (6.3–8.2)
≥25 years	62/1401	4.4 (3.4–5.7)	22/434	5.1 (3.3–7.7)	25/831	3.0 (2.0–4.5)	58/2469	2.3 (1.8–3.0)	167/5136	3.3 (2.8–3.8)
HSV2 status at enrolment										
Positive	107/1515	7.1 (5.8–8.5)	36/528	6.8 (4.9–6.0)	35/810	4.3 (3.1–6.0)	78/2327	3.4 (2.7–4.2)	256/5180	4.9 (4.4–5.6)
Negative/Equiv	39/826	4.7 (3.4–6.5)	33/574	5.8 (4.1–8.1)	15/510	2.9 (1.8–4.9)	39/1031	3.8 (2.7–5.2)	126/2942	4.3 (3.6–5.1)

Week 52 analysis. Values are # HIV infections/# person-years; HIV incidence per 100 person-years (95% confidence interval (CI)).

^a^Comparing rates across four HC groups: logrank *P* < 0.001.

### Risk factors for HIV infection

The following baseline factors were found to be independently associated with HIV risk: age, centre and HSV-2 result. Time-updated covariates found to be independently associated with HIV were vaginal discharge, chlamydia, bacterial vaginosis and HSV-2. Although not associated with HIV, the randomized gel group was also included in the baseline and time-updated covariate adjusted HIV outcome models. Neither frequency of sex nor reported condom use was independently associated with HIV outcomes in this dataset (neither at baseline nor follow-up), but were included in the baseline and time-updated covariate adjusted HIV outcome models.

### Pregnancy in follow-up

In total 908 women became pregnant in follow-up: 122 (5%) among those using DMPA at baseline; 58 (5%) for baseline Net-En users; 247 (18%) for baseline OC users and 481 (13%) for baseline no HC users. Pregnancy was included in the time-updated covariate adjusted model, although it was not found to be a significant predictor of HIV risk: HR = 1.21 (95% CI 0.75–1.95).

### Effect of HC on risk of HIV

Table IV(a) shows the effect of HC on risk of HIV acquisition. The univariable unadjusted effects of *baseline* HC use, compared with no HC, were HR = 1.79 (95% CI 1.41–2.28) for DMPA, 1.80 (1.34–2.43) for Net-En and 1.09 (0.78–1.52) for OC. After adjusting for baseline factors, the effects for DMPA and Net-En reduced to 1.38 (1.07–1.78) and 1.18 (0.86–1.62), respectively, while the OC effect was similar to the unadjusted effect, 0.97 (0.68–1.38).

**Table IV:** Estimated effect of HC on HIV acquisition.

(a)	Univariable model	Baseline covariate adjusted model^a^	Univariable model	Baseline covariate adjusted model^a^	Time-updated covariate adjusted model^a^	
Contraception	Baseline	Baseline	Current	Current	Current	
DMPA	1.79 (1.41–2.28)	1.38 (1.07–1.78)	1.86 (1.43–2.41)	1.33 (1.01–1.75)	1.45 (1.09–1.93)	
Net-En	1.80 (1.34–2.43)	1.18 (0.86–1.62)	1.81 (1.32–2.47)	1.07 (0.77–1.50)	1.20 (0.84–1.69)	
OC	1.09 (0.78–1.52)	0.97 (0.68–1.38)	1.06 (0.77–1.47)	0.84 (0.60–1.18)	0.90 (0.63–1.26)	
No HC	1	1	1	1	1	
LRT *P*-value^c^	<0.001	0.062	<0.001	0.020	0.008	
**(b)**	**Univariable model**	**Baseline covariate adjusted model^a^**				**Inverse probability weighted model^b^**
Contraception	Baseline	Baseline				
DMPA	2.29 (1.68–3.11)	1.46 (1.05–2.04)				1.49 (1.06–2.08)
Net-En	2.43 (1.69–3.48)	1.33 (0.89–1.99)				1.31 (0.86–1.99)
OC	0.99 (0.87–1.97)	1.00 (0.64–1.58)				1.00 (0.62–1.61)
No HC	1	1				1
LRT *P*-value^c^	<0.001	0.085				0.067

Values are estimated hazard ratios (95% CI) (a) Week 52 analysis (based on 382 HIV infections); (b) Week 52 analysis, censored for changing HC and pregnancy (based on 282 HIV infections).

^a^Models adjusted for age, centre, HSV-2 result, chlamydia result, bacterial vaginosis, vaginal discharge, frequency of sex and condom use, pregnancy (time-updated model only) and randomization.

^b^Weights for inverse probability weighted (IPW) model computed from separate, within centre, multinomial logistic regression models for use of HC and censoring in follow-up and pregnancy, including the variables listed above (except pregnancy) and education, employment status, genital ulcers, genital itching, ectopy and other sexually transmitted infection results. Weights, mean (range): 1.00 (0.14–4.09).

^c^Likelihood ratio test *P*-values comparing the effects of the contraceptive methods.

Results from models measuring the effect of *current* HC use were broadly similar to the baseline HC effects. Including time-updated covariates slightly increased the effect for DMPA: HR = 1.45 (95% CI 1.09–1.93) and Net-En, HR = 1.20 (95% CI 0.84–1.69). This model included sexual behavioural factors (sexual frequency and condom use) and pregnancy.

Table IV(b) shows the estimated hazard ratios where data were censored at the point of the first change in HC (stopping, starting or switching) or pregnancy. Full details of the causal model are given in the Supplementary data. Results from fitting the same univariable and baseline adjusted models as were fitted on the full dataset produced larger effects in this reduced dataset. However after adjusting for the artificial censoring and censoring for pregnancy (as well as natural censoring), the weighted model gave similar results to the previous adjusted models on the full dataset, with the DMPA effect remaining consistently significant and elevated in all models, causal HR = 1.49 (95% CI 1.06–2.08).

Interactions were tested on the *baseline* effect models on the full dataset. None of the pre-specified interactions were significant, with the results consistent across age groups (*P* = 0.71), HSV-2 status at enrolment (*P* = 0.61) and centre (*P* = 0.59).

Our findings were also robust to a number of sensitivity analyses. Restricting the analyses to those who never missed more than two consecutive visits (79%) gave similar results to the full dataset. Similarly, restricting to those who reported not using a condom at least twice in follow-up (82%) also gave similar results. The HC effects also remained unaffected by creating a separate category for those reporting using a condom specifically for family planning purposes (from the no HC group). There was no difference in the risk of HIV for those reporting using a condom for family planning compared with the remainder of the no HC group: HR = 0.97 (95% CI 0.64–1.46) and effects for DMPA, Net-en and OC were similar to the main findings. Finally, restricting the analysis to those who were not on HC at screening (up to 6 weeks prior to enrolment) also gave similar results (data not shown).

## Discussion

We found a modest elevated risk (with a relatively wide CI) of HIV acquisition for DMPA use that we were not able to explain from the measured confounders in this dataset. Our effect estimate for Net-En suggests a moderately increased risk of HIV but this was not statistically significant. There was no evidence of an effect of OC on risk. This is the largest single study to date to report on this question and the results are similar (in terms of magnitude of effect sizes) to those found in three recently reported studies (Heffron *et al.*, 2012; Morrison *et al.*, 2012; McCoy *et al.*, 2013). Not all studies have found an association between use of injectable HC and increased risk of HIV, and because previous studies have used a range of methods, sample sizes and data of variable quality, as highlighted in a recent systematic review (Polis and Curtis, 2013), these factors could in part explain the variable findings. As far as possible we followed the recommended approaches to analyses for investigating this question (Polis *et al.*, 2013).

Two particular strengths of this dataset were 4-weekly reporting of contraception and condom use and the ability to distinguish between DMPA and Net-En. Recent research has suggested possible differential biological pathways of different progestins used in contraception that may have a role in increasing susceptibility to HIV acquisition (Tomasicchio *et al.*, 2013). Although our results for Net-En were less consistent, the effects were consistently significant for DMPA.

The OC group is prone to miss-specification as user-dependent methods are commonly associated with lower adherence, backed up in this dataset by the higher pregnancy rates observed in the OC group compared with the other groups. Although some injectables were delivered in the study clinics, there was still substantial reliance on self-report from the participant and the timing of each injection could not be determined in this dataset. The sexual behavioural data, including condom use, are similarly prone to misreporting. In this dataset, although condom use was not associated with a lower risk of HIV acquisition, it was associated with lower risk of pregnancy (HR = 0.73, 95% CI (0.63, 0.84) among those not using injectables). While we acknowledge these limitations inherent in the dataset, we found our results to be robust to a number of different sensitivity analyses including an analysis which excluded users of condoms for family planning purposes from the no HC group. Importantly our findings were consistent across centres. A unique feature of the dataset is that the Ugandan site enrolled sero-discordant couples where women knew they were at risk of HIV infection, which may not have been the case elsewhere. Similar to recent studies we found no evidence of effect modification for age or HSV2 status (Heffron *et al.*, 2012; McCoy *et al.*, 2013).

A main limitation of the study was that it was a secondary analysis of data from a study that was not designed to investigate this question. The causal effect model aimed to emulate results that would be seen in a RCT in which adherence to 52 weeks of HC use was perfect. We estimated the effect of 52 weeks of HC use by type by artificially censoring and weighting for first change in baseline HC, assuming the adjustment for baseline covariates appropriately allowed for differences in baseline HC choice. By only including follow-up time on baseline HC and conservatively using the date of first positive HIV test as date of infection, we avoided ambiguity regarding the type of HC use at infection. Our hazard ratios can be interpreted as average effects over 52 weeks.

Despite our best efforts, we cannot exclude residual confounding to explain the effect of DMPA, and a RCT of HC methods is currently being planned to address this (Morrison and Nanda, 2012). However, time-dependent confounding could still occur, with switching and pregnancy likely. Further, it is highly probable that there will be differential condom use if the WHO recommendations are followed, and measuring this, together with sexual behaviour, would rely on self-report. These methodological concerns have raised questions regarding the value of such a trial (Gollub and Stein, 2012; Morrison and Nanda, 2012; Ralph *et al.*, 2013, 2014; Cates, 2014), and the investigators acknowledge the threat to the quality of data generated. In parallel to the planning of a randomized trial, an individual patient data meta-analysis is underway, with >1800 incident HIV infections in the combined 18 longitudinal datasets, and this will determine the most precise estimates for DMPA and Net-En so far.

WHO guidelines use the GRADE scheme to evaluate the evidence taking account of study quality, the size of the effect and precision around the estimate. A WHO Expert committee then reviews the GRADEd evidence and considers the risk benefit considerations of changing current guidance on the use of DMPA in high HIV prevalence settings, with particular attention being paid to any resulting rise in unintended pregnancy and associated maternal mortality and morbidity, versus a reduction in HIV morbidity and mortality (Butler *et al.*, 2013). A modest increase in risk for HIV acquisition does not justify withdrawal of DMPA where this is the only available HC, and overall HIV incidence is low. However this is not the case in South Africa, where HIV incidence amongst women remains high, and alternative HC methods are available. The current recommendation that women at risk of HIV use condoms as well as DMPA is of little use to a large proportion of women using DMPA as this study shows. The results of this study and other new data soon to be published should be urgently reviewed by WHO to see if current recommendations require modification.

What is certain is that the majority of women who seek a long-acting contraceptive method living in countries with a high prevalence of HIV will continue to have little choice other than DMPA for the foreseeable future. This is not an acceptable situation. A change in the WHO recommendations would undoubtedly accelerate the provision of alternative long-acting contraceptives (such as intrauterine contraceptive devices or hormonal implants) as well as the introduction of new methods. However, the time has already come for greater investments by the HIV and family planning fields to increase efforts towards this programmatic goal.

## Supplementary data

Supplementary data are available at http://humrep.oxfordjournals.org/.

## Authors’ roles

A.M.C. performed the statistical analysis and wrote the first draft of the manuscript. D.F. and A.M.C. worked on the methods for fitting the casual effects models. All authors contributed with substantive revisions to subsequent drafts. All authors read and approved the final manuscript.

## Funding

MDP is a partnership of African and European academic/government institutions with commercial organizations, which is funded by the UK Government (Department for International Development and Medical Research Council), with support from International Partnership for Microbicides and European Developing Countries Clinical Trials Partnership. The funders had no role in study design, data collection and analysis, decision to publish, or preparation of the manuscript. Funding to pay the Open Access publication charges for this article was provided by the Medical Research Council.

## Conflict of interest

None declared.

## Supplementary Material

Supplementary Data
