# Supplementary material for: Injectable and oral contraceptives and risk of HIV acquisition in women: an analysis of data from the MDP301 trial
Source: Hum Reprod. 2014 May 16;29(8):1810–7. doi: 10.1093/humrep/deu113 (PMC4093991; doi:10.1093/humrep/deu113)
Supplement: Supplementary Data [file supp_29_8_1810__index.html]

Injectable and oral contraceptives and risk of HIV acquisition in women: an analysis of data from the MDP301 trial — Supplementary Data 

# Injectable and oral contraceptives and risk of HIV acquisition in women: an analysis of data from the MDP301 trial

## Supplementary Data

Supplementary Data

**Files in this Data Supplement:**

- Supplementary Data - pdf file
