# Supplementary material for: Injectable and oral contraceptives and risk of HIV acquisition in women: an analysis of data from the MDP301 trial
Source: Hum Reprod. 2014 May 16;29(8):1810–7. doi: 10.1093/humrep/deu113 (PMC4093991; doi:10.1093/humrep/deu113)
Supplement: Supplementary Data [file supp_deu113_deu113_suppl_data.pdf]

Human Reproduction, Vol.0, No.0 , 2014
doi:10.1093/humrep/deu113

human reproduction

SUPPLEMENTARY DATA

Statistical methods for fitting weighted Cox models to estimate causal hazard ratios

Pooled logistic regression models were used to approximate weighted Cox models (due to software limitations) to estimate causal hazard ratios for 52 weeks HC use by type versus no HC use in non-pregnant women. Women were 'artificially censored' at the first of deviation from baseline HC or first positive pregnancy test. Natural censoring also occurred where women were lost to follow-up. The joint probability of remaining uncensored for change in HC use, for pregnancy and for loss to follow-up was estimated conditional on prognostic factors to obtain inverse-probability-of-censoring weights (*weighting* models). Then the association between baseline HC type and HIV diagnosis was estimated in a logistic regression model (*outcome* model) including baseline covariates weighted using the inverse-probability-of-censoring weights.

Data were organized into 4-weekly visits. Baseline factors were those at enrolment. Each 4-week interval, k, was bounded by $(t, t+28]$ with new intervals beginning at $t = 0,28,56,\ldots$ days after baseline corresponding to scheduled clinic visits.

Baseline covariates in weighting and outcome models were age, centre, education, employment status, randomized group, sexual frequency, condom use, genital findings, BV result, HSV-2 result and other STI results. Time since enrolment was adjusted for using restricted cubic splines with knots at 10th, 50th and 90th percentiles (8, 28 and 48 weeks).

Let $D(t)$ denotes an indicator of a positive HIV test during interval t. The outcome model was defined as follows:

$$\begin{aligned}\mathrm{logit}(P(D(t=1)|D(t-1)=0, N(t=0), CP(t=0), C(t=0), V)\\ = \theta_0(t) + \theta_1' V + \theta_2' X\end{aligned}$$

where $N(t)$ denotes an indicator of artificial censoring for deviating from baseline contraception in interval t (starting, stopping or changing type of HC), $CP(t)$ denotes pregnancy in interval t and $C(t)$ denotes loss to follow-up in interval t. V is a vector of baseline factors as described above and X is a categorical variable for baseline contraception (no HC, DMPA, Net-En, OC).

A subject in the risk set for interval t ($(t-28, t]$) was weighted by the inverse probability weight $SW^N(t) \times SW^{CP}(t) \times SW^C(t)$. The stabilized inverse probability weight $SW^N(t)$ for interval t was defined as

$$SW^N(t) = \frac{\prod_{k=1}^{t} P[(N(k)=0|N(k-1)=0,\ CP(k)=0,\ C(k)=0,\ V)}{\prod_{k=1}^{t} P(N(k)=0|N(k-1)=0,\ CP(k)=0,\ C(k)=0,\ \bar{L}(k-1))}$$

where $\bar{L}(t)$ is history of time-dependent confounders at/prior to the end of interval t, including baseline factors, V. Time-dependent variables included in $\bar{L}(t)$were sexual frequency, condom use, genital findings, BV result and HSV-2 result and other STI results. The stabilized inverse probability weight $SW^{CP}(t)$ for interval t was defined as

$$SW^{CP}(t) = \frac{\prod_{k=1}^{t} P[(CP(k)=0|CP(k-1)=0,\ N(k-1)=0,\ C(k)=0,\ V)}{\prod_{k=1}^{t} P(CP(k)=0|CP(k-1)=0,\ N(k-1)=0,\ C(k)=0,\ \bar{L}(k-1))}$$

The stabilized inverse probability weight $SW^C(t)$ for interval t was defined as

$$SW^C(t) = \frac{\prod_{k=1}^{t} P[(C(k)=0|C(k-1)=0,\ N(k-1)=0,\ CP(k-1)=0,\ V)}{\prod_{k=1}^{t} P(C(k)=0|C(k-1)=0,\ N(k-1)=0,\ CP(k-1)=0,\ \bar{L}(k-1))}$$

The probability of censoring for change in contraceptive use was estimated within strata defined by centre and baseline contraceptive type. For users of HC we fitted a multinomial logistic regression model with outcomes, no change (uncensored), stopped HC (censored) and changed HC type remaining on HC (censored). For non-users of HC we fitted a multinomial logistic regression model with outcomes, no change (uncensored), initiated DMPA (censored), initiated Net-En (censored) and initiated oral contraception (censored).

The probability of censoring for pregnancy and similarly, the probability of censoring for loss to follow-up were estimated within strata defined by centre from a logistic regression model including baseline contraception in addition to other baseline and time-dependent covariates.

We censored at pregnancy since pregnancy cannot be included as a standard time-dependent covariate in weighting models for deviation from baseline contraception; the probability of HC use during pregnancy is 'zero' (at least for initiation) leading to structural violation of positivity assumption when using inverse probability weighting for exposure. Results are therefore interpretable as risks associated with HC use in non-pregnant women.
